# The effect of digital health intervention in promoting healthy behavior: A systematic scoping review on strategies to prevent non-communicable diseases

**DOI:** 10.1016/j.dialog.2025.100258

**Published:** 2025-11-14

**Authors:** Zahroh Shaluhiyah, Shabrina Arifia Qatrannada, Aditya Kusumawati, Mohammad Shahgahan Miah

**Affiliations:** a Department of Health Promotion and Behavioral Sciences, Faculty of Public Health, Diponegoro University, Semarang, Indonesia; b Department of Anthropology, Shahjalal University of Science and Technology, Sylhet, Bangladesh

**Keywords:** Digital health, Social media, Noncommunicable disease, Healthy lifestyle, Systematic scoping review

## Abstract

**Background:**

Digital health interventions are increasingly used to prevent non-communicable diseases (NCDs) by promoting healthy behaviors, yet evidence on which digital features are most effective remains fragmented. This systematic scoping review aimed to (1) identify the most commonly used types of digital interventions and their target populations for NCD prevention, (2) examine the primary objectives associated with each digital feature, and (3) assess their effectiveness in improving knowledge, attitudes, and behaviors.

**Methods:**

A comprehensive search was conducted across seven databases (PubMed, ScienceDirect, Scopus, JSTOR, Medline, CINAHL, and ProQuest). Following PRISMA guidelines, 20 peer-reviewed studies published between 2018 and 2024 met the inclusion criteria. Data were extracted on digital features, intervention objectives, and outcomes related to knowledge, attitudes, and behaviors.

**Results:**

Messaging platforms were the most common (*n* = 7), followed by gamification, mobile applications, and wearables. Most interventions aimed to promote behavior change, especially those using mobile apps, messaging, and wearable tools. Similar digital features served different objectives depending on content design. Across studies, knowledge outcomes improved by 10–95 %, attitudinal outcomes by 2–40 %, and behavioral outcomes by 4–95 %. Messaging platforms showed the largest improvements in both knowledge and behavior, while gamification yielded moderate gains. Aligning digital tools with target users and intended outcomes enhanced overall impact.

**Conclusions:**

Messaging platforms and mobile applications emerged as the most frequently used and effective digital features for NCD prevention. Multi-feature interventions and platform–outcome alignment appear crucial to maximize effectiveness in digital health programs promoting healthy behavior.

## 1 Introduction

### 1.1 Background

Non-communicable diseases (NCDs) account for approximately 73 % of global deaths, or 41 million fatalities annually [1]. The rising prevalence of cardiovascular disease, diabetes, hypertension, and obesity highlights the persistent and widening global burden [[2], [3], [4], [5]]. This trend increasingly affects younger populations, reflecting prolonged exposure to behavioral risk factors such as unhealthy diet, physical inactivity, and disrupted sleep patterns [6,7], which in turn contribute to major social and economic consequences [8].

Modifiable lifestyle behaviors such as excessive consumption of junk food, sedentary routines, and inadequate sleep are major contributors to the NCD burden. Approximately 70 % of the global population regularly consumes unhealthy foods, which increase the risk of obesity, high cholesterol levels, and hypertension [9]. Sedentary work environments and increased reliance on digital technology further limit physical activity, with nearly 31 % of adults globally not meeting the recommended activity levels, equating to 1.8 billion individuals by 2022 [10,11]. Inactivity significantly increases the risks of heart disease, diabetes, and stroke [12]. Moreover, irregular sleep, often driven by excessive screen time, contributes to metabolic syndromes and other chronic conditions [13].

Owing to this escalating crisis, the integration of digital health technologies offers a promising avenue for addressing lifestyle-related NCD risks [14]. Digital health interventions, such as mobile apps, wearables, and online platforms, have been widely used to promote healthier behavior with widespread access to mobile devices and internet connectivity [15] These tools support self-monitoring, remote consultations, and adherence to lifestyle recommendations while also expanding access to underserved populations.

Systematic scoping reviews have shown that mobile health applications can effectively increase physical activity and support weight loss in various populations [16]. Similarly, wearables aid in tracking the diet and physical activity [17]. Digital health intervention is recognized as a cost-effective strategy to improve health outcomes while enhancing service delivery in line with the Sustainable Development Goals (SDGs) [18,19]. These technologies provide personalized guidance, real-time monitoring, and continuous user engagement and can be adapted to diverse sociocultural contexts [20] [21].

While meta-analyses are widely regarded as the gold standard for synthesizing evidence because they provide pooled effect sizes and quantitative estimates of intervention effectiveness, their application requires homogeneity in study designs, populations, and outcome measures. In the field of digital health interventions for NCD prevention, however, existing studies are highly heterogeneous in terms of platforms used, targeted behaviors, outcome indicators, and study designs. This heterogeneity limits the feasibility of conducting a robust meta-analysis at the current stage. Therefore, a scoping review was chosen to systematically map the breadth of available evidence, capture the diversity of digital intervention features, and identify knowledge gaps that can inform and guide future meta-analyses. Unlike meta-analyses, scoping reviews aim to map the scope, nature, and extent of existing evidence rather than quantify effect sizes.

The improvement of digital health intervention delivery has been identified as a critical strategy for addressing NCD risk factors and promoting well-being aligned with the United Nations Sustainable Development Goals (SDGs) [18]. Furthermore, the cost-effectiveness of digital health interventions has been highlighted, with studies emphasizing their potential to enhance safety, efficacy, and quality of care, while reducing healthcare costs [19]. Personalized health information, real-time monitoring, and continuous engagement have been delivered through digital platforms, contributing to improved health outcomes at a relatively low cost [20]. Digital interventions have also been recognized for their scalability across diverse populations and adaptability to local cultural and contextual needs [21]. However, there is a lack of comprehensive systematic scoping reviews summarizing digital strategies for promoting healthy lifestyles and evaluating their effectiveness in preventing NCD [21].

### 1.2 Research objectives and research questions

This scoping review focuses on digital interventions for NCD prevention. It aims to map existing evidence and address the following research questions:

1. Which types of digital interventions are most commonly used, and which target populations are they best suited for?
2. How do different digital features align with their intended objectives in promoting healthy behaviors?
3. What patterns can be observed in the effectiveness of these digital features in improving knowledge, attitudes, and behaviors related to NCD prevention?

## 2 Methodology

This systematic scoping review examined the characteristics, delivery mechanisms, and outcomes of digital health interventions using social media and other digital platforms to prevent NCDs and promote healthy lifestyles. This review aimed to explore and synthesize evidence from primary research that utilized various digital strategies to evaluate their influence on health-related behaviors and outcomes. Key characteristics of digital interventions were identified following a rigorous process of literature screening, data extraction, and critical quality appraisal. Although it was not a meta-analysis, this review adhered to the PRISMA 2020 guidelines for the selection process.

### 2.1 Study design

This systematic scoping review aimed to evaluate the role of digital health interventions in promoting healthy lifestyle behaviors and preventing noncommunicable diseases (NCDs). The primary objective was to assess how these interventions influence key individual-level outcomes, namely, health-related knowledge, attitudes, and behaviors. To ensure methodological rigor and comparability across studies, this review exclusively included experimental studies, including trials (RCTs) and quasi-experimental designs. Studies that employed observational, qualitative, or mixed-methods approaches were excluded. Notably, this review does not employ meta-analytic techniques; rather, it synthesizes experimental evidence to identify mechanisms and outcomes associated with digital intervention strategies.

### 2.2 Search strategy

The formulation of the search strategy followed a structured process consisting of the following steps.

1. Core keywords were generated based on research questions and insights from previous relevant studies.
2. Synonyms, related terms, and spelling variations were identified systematically to ensure comprehensiveness.
3. The Boolean operator OR was used to connect synonymous terms within each concept.
4. Different conceptual groups were combined using the Boolean operator AND to construct the final search string.

### 2.3 Database selection

To ensure a comprehensive and replicable review process, a literature search was conducted across seven prominent academic databases: PubMed, ScienceDirect, Scopus, JSTOR, MEDLINE, CINAHL, and ProQuest. These databases were selected because of their extensive coverage of interdisciplinary health, medical, and social sciences literature relevant to digital health interventions and non-communicable disease (NCD) prevention. The search period was restricted to publications published between 2018 and 2024, to reflect the most recent advancements in digital health technologies. The final database search was completed on June 10, 2024.

Search strings were developed by combining relevant keywords using Boolean operators (AND, OR, and NOT) to maximize sensitivity, while maintaining specificity. Each search string was tailored to the syntax of the respective databases and review articles (including systematic scoping reviews, scoping reviews, and meta-analyses) were excluded to focus solely on the original intervention studies. The refined queries aimed to capture studies addressing digital or social media-based interventions to promote health behavior changes related to NCDs. Table 1 presents the final search strings and number of articles retrieved per database after applying the initial filters and exclusion criteria. A comprehensive search strategy was developed using controlled vocabularies and synonyms (e.g., mHealth, eHealth, digital intervention). The full search strings, synonym generation, and pre-search validation process are provided in Supplementary File Table 1 to ensure transparency and reproducibility.

**Table 1:** Search input.

Database	Search String
PubMed	(“social media” OR “digital health”) AND (intervention) AND (“noncommunicable disease”) AND (“health promotion”) NOT (review)
ScienceDirect	(“intervention for noncommunicable disease”) AND (“social media” OR “digital health”) NOT (review)
Scopus	(“health promotion” OR “health education”) AND (intervention OR strategy) AND (“noncommunicable disease” OR “degenerative disease”) AND (“social media” OR “digital health”) NOT (review)
JSTOR	((“social media” OR “digital health”) AND (intervention) AND (“noncommunicable disease”)) NOT (review) AND (“public health” OR “degenerative disease”)
Medline	(“social media” OR “digital health”) AND (intervention OR strategies) AND (“noncommunicable disease” OR “degenerative disease”) NOT (review OR meta-analysis)
CINAHL	(“social media” OR “digital health”) AND (intervention OR strategies) AND (“noncommunicable disease” OR “degenerative disease”) NOT (review OR meta-analysis)
ProQuest	(“health promotion”) AND (intervention) AND (“noncommunicable disease”) AND (“social media” OR “digital health”) NOT (review OR scoping review)

### 2.4 Criteria for inclusion and exclusion

Studies were included if they met the following eligibility criteria: (1) published between 2018 and 2024 to ensure recency and relevance to current digital health landscapes; (2) experimental studies with clearly described interventions and measured outcomes, where outcomes could be assessed using objective indicators (e.g., BMI, blood pressure, step counts from devices) or self-reported measures (e.g., knowledge, attitudes, or health behaviors); (3) the intervention explicitly aimed at preventing non-communicable diseases (NCDs) or promoting a healthy lifestyle, such as increasing physical activity, improving diet, reducing smoking, or managing screen time; (4) utilized digital features or components (e.g., apps, wearables, messaging, online platforms) as part of the intervention delivery; (5) the intervention tools or platforms were accessible to the general population or specific communities (not limited to clinical or hospital-based interventions); and (6) full-text articles were available in English and retrieved through academic databases, as English remains the dominant language of high-impact scientific publications. While this decision may introduce a risk of language bias, it was deemed necessary to ensure consistency, accessibility, and feasibility of appraisal within the research team's expertise.

Studies were excluded if (1) they were systematic scoping reviews, scoping reviews, meta-analyses, protocols, editorials, or commentaries; (2) they did not contain empirical data or measurable outcomes related to behavior, knowledge, or attitude change; (3) the intervention did not include any digital components, or only discussed technology use without implementing it in the intervention; (4) the primary focus was on the management of existing NCDs rather than prevention or health promotion; (5) the digital tools used were not described in sufficient detail to assess their features or relevance; and (6) the study population was not clearly defined or the study design did not provide adequate methodological rigor (e.g., unclear sample, no control/comparison group, or unvalidated outcomes).

Additionally, studies were excluded if the intervention focused solely on clinical treatment adherence without addressing behavioral changes or health education objectives or if the target outcome was not aligned with any lifestyle-related risk factors of NCDs.

### 2.5 Study selection

The study selection process consisted of three stages. Initially, duplicate articles were identified and removed using Mendeley software. In the first stage, titles and abstracts were evaluated to determine relevance based on the predefined inclusion and exclusion criteria. Subsequently, abstract screening was performed, and studies focused on digital or social media-based interventions for health promotion targeting NCD, peer-reviewed, and published in English were included. The exclusion criteria were systematic scoping reviews, meta-analyses, general reviews, protocols, or studies not directly related to degenerative diseases or digital and social media interventions. Any disagreements between reviewers were resolved through discussion and consensus; a third reviewer was consulted when necessary.

**Fig. 1:**
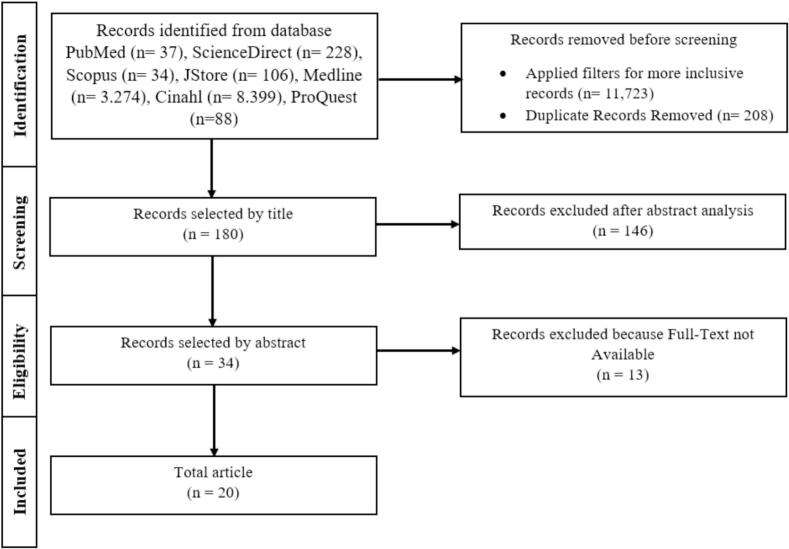
Sampling

This study identified 132,121 articles from seven source databases. After removing duplicates, a total of 483 articles were screened. Title screening subsequently reduced the pool to 180 articles distributed across the following databases: CINAHL (40), JSTOR (39), Medline (51), ProQuest (10), PubMed (15), ScienceDirect (14), and Scopus (11). The abstract screening process further narrowed down the selection process to 34 articles. After a final assessment of availability, 20 articles were retained for full review (Fig. 1).

In this review, the classification of digital intervention features was adapted based on the digital intervention frameworks of WHO and refined through internal discussions with the research team to ensure clarity and applicability [22]. Digital interventions were grouped into seven categories: messaging platforms, social media platforms, mobile apps, gamification and interactive media, wearable and monitoring tools, video-conferencing tools, and web-based tools (see Table 2). This categorization was intended to simplify interpretation by aligning each digital modality with its primary mode of user interaction and functionality, thereby facilitating a comparative analysis across studies.

**Table 2:** Outcome categorization for digital intervention.

**Digital Features**	**Description**	**Examples**
Messaging Platforms	One-way or two-way text-based communication to deliver information	SMS, WhatsApp, LINE, Telegram
Social Media Platforms	Social networking sites to disseminate educational content & community engagement.	Instagram, Facebook, TikTok, Twitter
Mobile Apps	Smartphone-based apps designed for behavior tracking, and goal setting.	Health tracking apps, calorie counters, mental health apps
Gamification & Interactive Media	Integration of game elements (e.g., points, rewards, challenges)	Nutrition games, quiz apps, interactive learning modules
Wearables & Monitoring	Wearable technologies that track real-time physical and physiological data	Smartwatches (e.g., Fitbit, Apple Watch), activity trackers, sleep monitors
Video Conferencing Tools	Real-time video platforms that enable direct interaction	Zoom, Skype, Google Meet
Web-based Tools	Website that provide structured & self-paced health education	Health education websites, e-learning platforms, digital risk calculators

### 2.6 Data extraction

The data extraction process was conducted independently by each author using the Atlas.TI 9. Various components of the studies were extracted and analyzed. The data chart included details such as author name, publication year, study design, country, objectives, sample size, population, type of digital health, setting, intervention duration, content, outcome measures, and outcomes as well as facilitators and barriers to the use of digital technology. The extracted data were validated by all authors, and any discrepancies were addressed through discussion, ensuring the accuracy and comprehensiveness of the information.

### 2.7 Data analysis

After the final selection of eligible articles, a structured analysis was conducted to synthesize findings relevant to the research questions. The analysis comprised the following stages.

#### 2.7.1 Categorization

The included studies were systematically categorized based on several key attributes: study origin, research objective, assessment methods, target population, intervention approach, and digital features employed. Additionally, each study was coded according to the type of intervention output targeted (knowledge, attitudes, or behaviors) and the magnitude of change reported (percentage increases or mean differences).

#### 2.7.2 Thematic analysis

A thematic analysis structured mapping process was employed to identify digital features associated with specific intervention domains. Studies were grouped according to the primary outcome targeted (knowledge, attitude, or behavior), and digital features, such as messaging platforms, social media platforms, mobile apps, gamification and interactive media, wearables and monitoring, video conferencing tools, and web-based tools, were analyzed for their prevalence and association with these outcomes.

#### 2.7.3 Comparative analysis

To identify recurring patterns, comparative analyses were conducted across studies with similar outcomes. Interventions that effectively improved knowledge were contrasted with those targeting attitudinal or behavioral changes to assess the consistency of digital mechanisms across different domains. This approach facilitated the identification of domain-specific strategies and potential transferability of intervention models.

#### 2.7.4 Interpretive synthesis

The findings were further synthesized to explore the underlying mechanisms through which specific digital features contribute to changes in knowledge, attitudes, and behavior. Where available, the theoretical justifications or explanatory frameworks provided by the original studies were examined to contextualize the observed outcomes.

By following this rigorous and systematic process, this review offers a comprehensive overview of how digital platforms are employed in behavior change interventions, emphasizing the mechanisms of engagement and observed outcomes in the prevention of non-communicable diseases.

### 2.8 Critical appraisal

Risk of bias assessment was conducted using the JBI Critical Appraisal tools for both randomized controlled trials (RCTs) and non-randomized experimental studies (Supplementary File Table 2, Table 3). Articles were classified as having a low risk of bias if their score was at least 70 %, moderate risk of bias if their score was 50 %–69 %, and high risk of bias if their score was less than 49 % [23,24]. Among the 12 RCTs evaluated, four studies (33 %) were rated as having a low risk of bias, six (50 %) as moderate, and two (17 %) as high. Common limitations of moderate-to-high-risk RCTs include unclear randomization procedures, lack of blinding, and incomplete follow-up reporting. Among the eight non-randomized studies, five (67 %) were judged to have a high risk of bias, two (22 %) moderate risk, and one (11 %) low risk. Most non-randomized studies demonstrated adequate reporting of intervention and outcome measurements, although several had unclear handling of confounders or participant inclusion criteria. To address the potential influence of high-bias studies, a sensitivity check was performed by re-examining effectiveness patterns after excluding studies rated as high risk. The overall direction and magnitude of reported improvements in knowledge, attitudes, and behaviors remained consistent, although the upper range of behavioral effects (≥90 %) was slightly attenuated when high-bias studies were removed. This suggests that while some individual estimates may be inflated by methodological limitations, the central conclusions of this review regarding the effectiveness of digital interventions for NCD prevention remain robust.

**Table 3:** Characteristics of the studies.

No	Authors	Study Origin	Assessment Method	Objective	Intervention target
1	Vizeshfar, 2021 [23]	Iran	RCT	Assesses effects of smartphone-based self-care training on elderly health.	Elderly from Jahandidegan Center
2	Young, 2021 [24]	Cambodia	Cluster RCT	Compares face-to-face and phone-based infant feeding counseling.	Mother with Children 6–24 months
3	Weiner, 2023 [25]	USA	Single arm feasibility trial	Evaluates peer-delivered remote intervention for cancer survivors	Female breast cancer survivor
4	Rahbar, 2024 [26]	Iran	Quasi Experimental	Examines effects of social media-based microlearning on diabetes self-care.	Diabetes type 2 Patients >18 years old
5	Schreiber, 2023 [27]	Austria	RCT	Measures effects of a smartphone-based intervention on health behavior.	Adolescents aged 18 years
6	Alhazmy, 2024 [28]	Saudi Arabia	Quasi	Assesses WhatsApp group impact on diabetes self-care and HbA1c.	Female adults with TM2 Diabetes
7	Ashley, 2021 [29]	Australia	Quasi	Develops an online program to improve food literacy via MedDiet.	18 years old
8	Jarrar, 2022 [30]	UAE	Controlled intervention	Measures digital education platform's effect on salt intake.	Healthy adults aged 20–40
9	Hesseldal, 2022 [31]	Denmark	RCT	Evaluate eHealth lifestyle coaching for obesity and diabetes.	BMI (30–45 kg/m2) and age (18–70 years).
10	Chang, 2022 [32]	Taiwan	Quasi	Evaluate a nutrition-focused game's impact on young children.	Preschool children 5–6 years old
11	Mack, 2020 [33]	Germany	RCT	Measures the impact of a dietary game on children's nutrition knowledge.	School children 9–15 years
12	Froome, 2020 [34]	Canada	RCT	Evaluate Foodbot Factory's effect on children's nutrition knowledge.	Schoolchildren
13	Pope, 2019 [35]	Minnepolis	RCT	Tests a smartwatch and social media program	College Students
14	Pontes, 2021 [36]	Portugal	RCT	Compares three educational strategies to improve vegetable intake.	Preschool Children
15	Peuters, 2024 [37]	Belgium	Quasi RCT	Assesses #LIFEGOALS mobile intervention on lifestyle and mental health.	Young adults or students
16	Vasil, 2023 [38]	Albanian	Prepost intervention survey	Measures behavior change from health-related apps in children.	Schoolchildren 11–15 years old
17	Brammall, 2024 [39]	Australia	RCT	Examines OptimalMe's effect on behavior change during preconception.	Women aged 18–44 years
18	Lee, 2020 [40]	Korea	Experimental	Evaluate e-Motivate4Change for metabolic syndrome prevention.	University Student
19	Chang, 2022 [41]	Taiwan	Quasi-Experimental	Examines social media-based nutrition education for truck drivers.	Trucks Drivers
20	Elling, 2020 [42]	Netherlands	RCT	Evaluates tailored e-cigarette information on smoking cessation decisions.	Adults (18+) who smoked within the past 7 days and aimed to quit within five years

## 3 Results

Following the application of the inclusion and exclusion criteria and screening of abstracts, 20 articles were deemed eligible for full review. Two authors independently conducted the review process for all selected studies, ensuring consistency in the quality assessment and research design standards. Any discrepancies in interpretation were resolved by discussion and consensus. The articles were subsequently organized based on their characteristics and thematic relevance to the research objectives (see Table 3).

### 3.1 Characteristics of the studies

This systematic scoping review included 21 studies conducted in diverse geographical settings, including Iran, the United States, Cambodia, Belgium, Norway, Saudi Arabia, Australia, Portugal, and several other countries, including Austria, the UAE, Denmark, Taiwan, Germany, Canada, Korea, Malaysia, Albania, and the Netherlands (Table 1). Methodological designs varied, including randomized controlled trials (RCTs), quasi-experimental studies, and pre-post-intervention surveys.

### 3.2 Synthesis of the studies

Among the 20 included studies, messaging platforms were the most frequently utilized digital modality, reported in seven studies. Gamification (five studies), wearables and monitoring tools (five studies), mobile apps (four studies), social media (two studies), video conferencing (two studies), and web-based tools (two studies) were used less commonly. These tools were often strategically aligned with user characteristics, messaging features such as SMS or WhatsApp were implemented among older adults, truck drivers, and patients with chronic conditions [23,24,26,41]; gamification was used in interventions targeting children and school-aged participants [[32], [33], [34]]; mobile apps and wearables appeared most often among university students or adults with specific behavioral goals [31,38,40,43]; and video conferencing or web-based platforms were deployed in contexts requiring either personal interaction or self-guided learning [25,29,35,39,42].

**Fig. 2:**
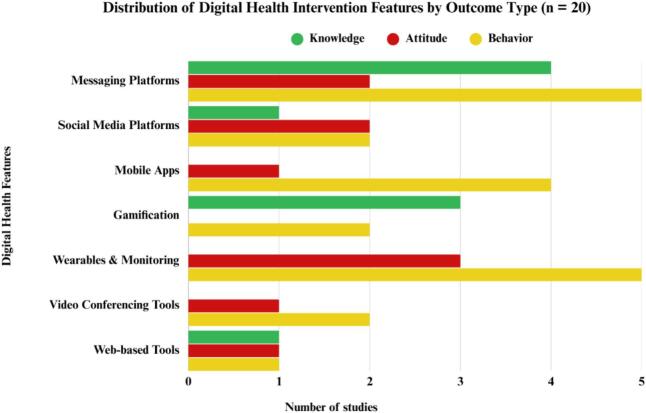
Digital features and their impact on each outcome.

Fig. 2 shows that most interventions were designed to influence behavior, with digital features such as mobile apps, messaging platforms, and wearable technologies serving as the primary vehicles for behavioral outcomes. Examples include smartphone-based training to improve self-care behaviors, counseling methods for feeding practices [26], and app-based lifestyle modification tools [35]. Gamification and messaging platforms were also used in knowledge-oriented interventions, particularly those aimed at nutrition and salt consumption, while web-based and social media tools were applied for both knowledge and attitudinal shifts [[32], [33], [34], [35], [36]]. Several studies have combined objectives to concurrently address knowledge, attitudes, and behaviors. Rahbar (2024) used social media–based microlearning to support diabetes self-care [26], Ashley (2021) integrated literacy and motivational components into dietary education [29], and Alhazmy (2024) leveraged WhatsApp groups to improve diabetes knowledge and behaviors [28].

Outcomes varied according to the intervention objective and digital modality (Table 4). The reported percentages represent absolute changes from baseline within individual studies, not pooled or relative measures. Percentage improvements reported in this review were calculated based on baseline-to-posttest differences as presented in each study. For example, a ‘95% improvement in knowledge’ indicates relative change from the pre-intervention baseline score to the final posttest score. Detailed definitions of effect indicators, including baseline measures, calculation methods, and significance levels, are provided in Supplementary Table 4. Knowledge gain was reported in 11 studies, ranging from 10 % to 95%. Messaging-based interventions reported the highest improvements, particularly in 95 % and 90 %, respectively [28,30]. Gamified features demonstrated moderate gains of 10–31 % [[36], [37], [38]], while mobile apps and web-based platforms yielded knowledge improvements of 40 % and 31 % respectively [31,42]. Attitudinal outcomes were reported in nine studies, with changes ranging from 2 % to 40 %. Improvements above 10 % were observed in interventions using wearables or video conferencing [25,38] with self-efficacy gains of up to 17 %. Messaging platforms showed smaller attitude improvements, between 2 % and 30 % [26,41]. Social media and web-based interventions have reached 40 % and 31 %, respectively [29,42]. Behavioral changes were reported in 17 studies with effect sizes ranging from 4 % to 95 %. The most substantial behavioral changes were associated with messaging platforms, which showed improvements between 25 % and 95 % in studies by Rahbar (2024) and Alhazmy (2024) [26]. Gamification yields behavioral gains of up to 93 %, particularly in dietary behavior interventions [36]. Combinations involving wearables, mobile apps, or video conferencing demonstrated behavioral improvements between 5 % and 64 % [25,[38], [39], [40],^43^], while social media–based interventions reported gains between 20 % and 40 % [29,35].

**Table 4:** Effects of digital technology-based promotion.

No	Authors	Digital Features	Intervention Approach	Intervention Output	Intervention Outcomes
1	Vizeshfar, 2021 [23]	Messaging Platforms	WhatsApp group delivered multimedia training and daily Q&A over three weeks, emphasizing engagement and feedback.	Behavior	General health, nutrition, and sleep quality increased, with absolute changes (Δ) ranging from 1.01 to 1.60, indicating a relative improvement of 4 %–7 % (*p* < 0.001).
2	Young, 2021 [24]	Messaging Platforms	PDH combined virtual and in-person sessions to enhance caregiving for underweight children.	Behavior	Underweight prevalence dropped by 12.8 % (*p* < 0.05)
3	Weiner, 2023 [25]	Wearable & Monitoring; Video Conferencing Tools	Zoom-based peer mentoring and Fitbit tracking supported physical activity in breast cancer survivors.	Attitude & Behavior	Physical activity & emotional support increased with changes (Δ) ranging from 4.3 to 37.2, indicating a relative improvement of 10 %–41 % (*p* < 0.001).
4	Rahbar, 2024 [26]	Messaging Platforms	A two-week WhatsApp microlearning used multimedia and expert talks for diabetes management.	Knowledge, Attitude, Behavior	Knowledge, Self-care, Exercise increased changes (Δ) ranging from 30 to 60, indicating a relative improvement of 13 %–45 % (p < 0.001).
5	Schreiber, 2023 [27]	Wearables & Monitoring	Daily diaries via smartphone tracked health behavior over three weeks with follow-ups.	Behavior	Health promoting behaviors increased changes (Δ) 0.43, indicating a relative improvement of 9 % (p < 0.001).
6	Alhazmy, 2024 [28]	Messaging Platforms	A multidisciplinary team used WhatsApp group to deliver daily interactive diabetes care content.	Knowledge & Behavior	self-care improved increased changes (Δ) ranging from 4 to 10, indicating a relative improvement of 25 %–95 % (*p* < 0.001).
7	Ashley, 2021 [29]	Social Media Platforms	A closed Facebook group taught the Mediterranean diet using visuals, recipes, and expert Q&A.	Knowledge, Attitude, Behavior	Fruit & vegetable consumption increased (Δ) ranging from 0.9 to 1.4, knowledge, attitudes, behavior 20 %–40 %
8	Jarrar, 2022 [30]	Messaging Platforms	WhatsApp and e-brochures repeated sodium-reduction messages; control group received none.	Knowledge	Salt reduction knowledge increased (Δ) ranging from 2.9 to 5, relative improvement 45 %–90 %
9	Hesseldal, 2022 [31]	Mobile Apps	App-based eHealth coaching included SMART goals, weekly monitoring, and expert feedback.	Behavior	Greater reductions in body weight (−4.5 kg vs −1.5 kg) and BMI (−1.5 kg/m^2^ vs −0.5 kg/m^2^) relative improvement 40 % (all *p* < 0.001)
10	Chang, 2022 [32]	Gamification	Interactive games taught preschoolers nutrition via hands-on activities and assessments.	Knowledge	Nutrition knowledge increased Δ = 8 indicatinga relative improvement 10 % (*p* < 0.002)
11	Mack, 2020 [33]	Gamification	A 3D game combined storytelling and motion to teach nutrition, activity, and stress relief.	Knowledge	Knowledge increased Δ = 16 indicating a relative improvement 30 % (*p* < 0.001), but no behavior change.
12	Froome, 2020 [34]	Gamification	Mobile apps offered gamified, daily nutrition education modules for children.	Knowledge	Knowledge increased Δ = 3.2 indicating a relative improvement 31 % (p < 0.001)
13	Pope, 2019 [35]	Wearables & Monitoring; Social media platforms	Wearables and Facebook posts promoted physical activity with motivational content.	Attitude & Behavior	Physical activity, dietary practices, self efficacy increased (Δ) ranging from 0.2 to 2, relative improvement 25 %–35 %.
14	Pontes, 2021 [36]	Gamification	Digital tools like games and storybooks encouraged children's vegetable intake.	Behavior	Vegetable intake increased (Δ) ranging from 0.7 to 6, relative improvement 40 %–93 % (*p* < 0.001)
15	Peuters, 2024 [37]	Mobile Apps	LIFEGOALS used videos, a chatbot, and self-regulation tools for healthy habit formation.	Behavior	Physical activity, positive mood, and sleep quality increased (Δ) ranging from 0.24 to 19.11, representing a relative improvement of approximately 5 %–30 %.
16	Vasil, 2023 [38]	Gamification; Mobile apps	Health application with quizzes and rewards supported health education in schoolchildren.	Behavior	Fruit and vegetable intake, toothbrushing, physical activity, and handwashing behaviors improved, with relative increases ranging from 9 %–24 % (*p* < 0.001)
17	Brammall, 2024 [39]	Web-based Tools, Video Conferencing	Zoom and email coaching guided dietary and physical activity improvements.	Behavior	Weight monitoring, preconception supplement use, alcohol reduction, and diet or physical activity behaviors increased, with relative improvements ranging from 35 % to 64 % across outcomes. (p < 0.001).
18	Lee, 2020 [40]	Mobile Apps; Wearables & Monitoring	A mobile app provided tailored health content, gamification, and real-time monitoring.	Attitude & Behavior	Significant increase in healthy lifestyle promotion (+18.4 points, ∼11 %) and self-efficacy (+162.4 points, ∼17 %), along with a decrease in BMI (−1.2 kg/m^2^) and cholesterol levels (−53.2 mg/dL). (p < 0.001).
19	Ibrahim, 2018 [48]	Messaging Platforms	Co-HELP integrated face-to-face group and individual education sessions with personalized behavior change plans. Digital elements were limited to phone-based follow-ups for monitoring and reinforcement.	Behavior	Physical activity, dietary habits, and self-efficacy improved (Δ) ranging from 18.9 to 154.2, representing relative improvements of approximately 12 %–16 %, while BMI and screen time decreased (Δ = −1.3 kg/m^2^ to −1.6 h/day).
20	Chang, 2022 [41]	Messaging Platforms	LINE messages, food photo logs, and incentives improved truck drivers' diet knowledge.	Attitude	Self-perceived susceptibility, severity, and self-efficacy improved (Δ = 0.07 to 0.28), representing relative improvements of approximately 2 %–10 %
21	Elling, 2020 [42]	Web-based Tools	Using the I-Change Model, advice was personalized by topic, based on user preferences.	Knowledge & Attitude	Knowledge scores improved (Δ = +1.13), representing a relative increase of approximately 31 %, while attitudes toward e-cigarettes also improved significantly (*p* < 0.01).

Subgroup analyses revealed clear patterns. Gamification and mobile apps were concentrated among children and adolescents, with outcomes on nutrition, hygiene, and physical activity. Messaging platforms and apps were most frequently used among adults with chronic conditions (e.g., diabetes, obesity), improving diet, physical activity, BMI, and self-care. Elderly populations, caregivers, and cancer survivors were more often reached through wearables or video conferencing, targeting physical activity, emotional support, and caregiving. By condition, messaging (particularly WhatsApp) was most effective for diabetes self-care [26,28], while mobile apps and web-based coaching dominated obesity and weight-management interventions [31,35,39]. Cancer-related studies utilized wearables and synchronous video conferencing [42], whereas cardiovascular risk programs favored app-based monitoring [40,43]. Regional trends also emerged: messaging platforms (notably WhatsApp) were prevalent in LMICs due to scalability and affordability, while apps and wearables were more common in HICs, reflecting broader technology access and feasibility for long-term monitoring.

### 3.3 Patterns of intervention complexity and their effectiveness

Across the 20 included studies, 13 implemented single-feature interventions (e.g., only messaging platforms, mobile apps, or gamification), while 7 studies employed multi-feature approaches that combined two or more digital tools (e.g., wearables with video conferencing, apps with monitoring tools). Single-feature interventions (*n* = 14) were largely delivered through messaging platforms (e.g., WhatsApp, LINE), gamification tools, mobile apps, or social media platforms. These interventions demonstrated substantial improvements in knowledge (Δ 10 %–95 %), modest-to-high gains in behavior (Δ 5 %–40 %), and, in some cases, attitude/self-efficacy (Δ 2 %–30 %). For instance, messaging platforms for diabetes care (Rahbar, Alhazmy, Jarrar) yielded strong knowledge and self-care outcomes (up to 95 % improvement), while gamification approaches in children significantly improved nutrition knowledge and vegetable intake (up to 93 %).

In contrast, multi-feature interventions (*n* = 7), such as wearables combined with video conferencing (Weiner, Brammall) or apps with monitoring tools (Lee, Pope, Vasil), generally produced broader and more sustained improvements across multiple outcome domains. These included not only knowledge and behavior change but also psychosocial dimensions such as emotional support and self-efficacy. The relative improvements in multi-feature interventions ranged from 9 % to 64 %, with particularly notable effects in weight management, physical activity, and lifestyle monitoring. For example, Brammall (2024) combining web-based coaching with Zoom sessions reported behavior improvements of 35 %–64 %, while Lee (2020) demonstrated significant reductions in BMI and cholesterol when integrating apps with real-time wearable monitoring. Overall, while single-feature interventions demonstrated strong effects in specific domains (particularly knowledge acquisition via messaging and gamification), multi-feature approaches appeared to yield more holistic and sustained benefits, especially when targeting complex outcomes such as weight reduction, chronic disease management, and self-efficacy. However, few studies conducted direct head-to-head comparisons between single- and multi-feature interventions, limiting the strength of evidence for synergistic effects.

## 4 Discussion

### 4.1 Alignment between digital features and target populations

The extensive use of messaging platforms in the reviewed interventions reflects more than technical simplicity. This reveals a strategic fit between the tool's characteristics and the needs of specific populations. Older adults and individuals with limited digital experience, for example, often prefer platforms such as SMS or WhatsApp, because of their ease of navigation and minimal interface complexity [44]. These platforms are “passive” in that users receive messages without the need to actively search for or interact with the content. This low-effort, low-cognitive-burden delivery allows information to reach users without time, sustained attention, or digital fluency [45]. Such characteristics make these platforms particularly well suited for users with constrained schedules, lower health literacy, or chronic conditions that reduce their capacity for active engagement. Beyond this “fit,” effectiveness also depends on technical accessibility. From a COM-B perspective, populations must not only have the capability (e.g., digital literacy and basic device skills) but also the opportunity (e.g., access to smartphones, stable internet, or sufficient data packages) to fully engage with interventions. Messaging platforms are effective partly because they minimize these barriers, requiring less digital skill and bandwidth compared to apps or video conferencing. This highlights that accessibility constraints, especially among the elderly or those in resource-limited settings, can shape whether an intervention is truly feasible.

In comparison, digital features such as gamification and mobile apps, are typically aligned with populations that are digitally literate and receptive to high-engagement formats [46]. These features particularly appeal to children, adolescents, and young adults who are familiar with interactive environments and are motivated by immediate feedback, progress tracking, and reward systems. For example, gamified interventions offer developmentally appropriate stimuli that foster learning and habit formation through structured play, such as points, levels, and challenges. Study from Chang (2022) used gamification to engage preschool children aged 5–6, a choice that might seem intuitive but reflects a deeper understanding of developmental psychology [32]. At that age, using fun or game-like formats helps them absorb healthy habits without even realizing they're learning [46]. Another study from Pontes (2021) and Mack (2020) deployed game-based learning to reach school-age children, banking on the idea that structured rewards, visual feedback, and narrative progression are not just entertaining but they're also cognitively and motivationally aligned with young learners [33,36]. Similarly, mobile apps and wearables are effective for users accustomed to self-monitoring tools, offering integration into daily routines for tracking steps, dietary intake, or other health behaviors [47]. Their users were not just passive recipients of content, but they also actively tracked behaviors, set goals, and monitored progress [37,40]. Hesseldal (2022) used an app with adults aged 18–70 who had a BMI between 30 and 45. This shows that even though tech skills may vary by age, people across generations can still engage with digital tools, especially when the app is simple and easy to use. However, these tools may demand higher baseline capability (comfort with app navigation, self-monitoring) and greater opportunity (regular internet, compatible devices), which could limit uptake in digitally disadvantaged groups.

Even less frequently used tools such as video conferencing and structured web-based modules show intentional population matching. Video conferencing tends to be employed in sensitive contexts, such as with cancer survivors or patients managing long-term conditions, where real-time dialogue can strengthen emotional support and foster accountability through face-to-face interactions [49]. These groups don't just need information, but they also benefit from talking to health facilitators, sharing with others, or being part of support groups [25,39]. Live sessions allow for real-time emotional connection and deeper relationships, which can't be fully replaced by pre-recorded or text-based tools [49]. Web-based tools suit autonomous adult learners, allowing flexible access to structured information that can be navigated without time pressure or social interaction. These users benefited from the flexibility to engage with content at their own pace, without the need for intensive guidance or synchronous interaction [42]. The ability to absorb information independently and revisit materials as needed made this format ideal for reflective, time-conscious adult users [50].

### 4.2 Purposeful use of digital features in driving specific outcomes

Beyond population targeting, digital features were purposefully selected to fulfill specific intervention objectives, particularly across behavior, knowledge, and attitudinal domains. Tools, such as mobile apps and wearables, are most commonly employed in behavior change interventions because of their ability to support dynamic goal setting, self-monitoring, and real-time feedback. These features translate abstract intentions into actionable and trackable behaviors. In studies such as Hesseldal (2022) and Schreiber (2023), users successfully integrated healthy practices into daily routines by receiving personalized cues, monitoring their progress, and responding to adaptive feedback [51]. These mechanisms align closely with the COM-B framework, as they enhance “capability” by supporting knowledge acquisition and self-regulatory skills, “opportunity” by embedding structured prompts within the user's environment, and “motivation” by reinforcing goals through feedback and progress tracking. Collectively, these elements establish the core conditions necessary for sustained behavior change, as proposed in the COM-B model [52].

Messaging platforms are also frequently used for behavioral change, but they operate via different mechanisms. Their strength lies in the timely, brief prompts that nudge users without demanding significant cognitive investment. Rather than intensive engagement, behavioral change here is reinforced through regular, contextually relevant cues that maintain salience over time [49]. Importantly, digital features should not only be viewed as content delivery mechanisms, but also as motivational drivers. For instance, gamification fosters intrinsic reward and enjoyment, while messaging systems can cultivate social accountability by reminding users that their progress is visible to peers or facilitators. These motivational pathways complement the capability- and opportunity-focused mechanisms, demonstrating how features directly contribute to sustaining engagement and change.

When interventions were aimed at enhancing knowledge, digital features that emphasize repetition, simplicity, and control over content exposure were favored. Messaging platforms, gamification, and web-based modules have been used to reinforce key information through messages or by engaging learning formats. [53] Game-based tools, such as those used by Mack (2020) and Froome (2020), provide interactive exposure to content that could improve recall and motivation. However, without pedagogical scaffolding such as feedback loops, progressive complexity, or opportunities for reflection, these tools sometimes lead to superficial learning [53]. Similarly, web-based platforms such as those used by Elling (2020) allow for self-paced exploration of content, making them well-suited for complex information delivery. However, their limited interactivity often makes them less effective in influencing attitudes or motivation unless combined with more emotionally resonant components. Taken together, these nuances suggest that effectiveness lies not only in “which feature is used,” but also in “how the feature is psychologically leveraged” to support motivation, reflection, or accountability.

Interventions that addressed multiple outcomes simultaneously were the most successful when they integrated features that enabled both cognitive and emotional engagement. Group-based tools such as WhatsApp and social media support this approach by enabling peer interaction, emotional support, and the co-construction of meaning [54]. In Ashley (2021) and Alhazmy (2024), for instance, digital spaces were used not only for delivering facts but also for building motivation and accountability through shared dialogue and collective experience. These findings highlight that the effectiveness of a digital feature is not intrinsic, but depends on how well it aligns with the intervention's psychological and behavioral objectives.

### 4.3 Explaining differential effectiveness across knowledge, attitudes, and behavior

Effectiveness across the knowledge, attitude, and behavior domains varied significantly, reflecting the distinct psychological demands of each outcome. Knowledge, being largely cognitive and factual, responded best to tools that offered clarity, repetition, and a low interaction burden. Messaging platforms, particularly those used in studies such as Alhazmy (2024) and Jarrar (2022), demonstrated high effectiveness in knowledge acquisition through the delivery of small, manageable content chunks. This “drip-feed” model reduced cognitive load, supported information retention, and allowed users with limited time, digital skills, or competing life demands to absorb content gradually without disengagement [55]. Such knowledge gains can occur rapidly even with short-term exposure, because the COM-B requirement is primarily cognitive capability rather than sustained motivation or opportunity.

Behavior change requires more active and structured support. Gamified tools and mobile apps are highly effective in this domain because they scaffold engagement through feedback systems, progress tracking, and daily challenges [56]. In one dietary intervention, gamification contributed to 93 % of behavior improvement, illustrating how motivational structures can reinforce and sustain new routines [36]. Wearables further strengthened this process by providing real-time physiological data and transforming abstract health advice into immediate personalized insight. Users can observe the consequences of their behavior, reinforce accountability, and make a path to improvement that is visible and actionable [57]. However, behavior change is rarely achieved through short-term interventions alone. From a COM-B perspective, behavior requires not only capability but also sustained motivation and long-term opportunity. This explains why interventions with longer duration and repeated reinforcement tend to produce more robust and durable behavior outcomes compared to brief programs.

Unlike knowledge or behavior, which can be reinforced through repetition and tracking, attitudes often require emotional resonance, social modeling, and reflective processes [58]. Interventions using video conferencing and social media were more effective in this area, as they created spaces for narrative sharing, empathy, and interactive discussion [59]. Weiner (2023) and Ashley (2021) showed how group-based dialogue promoted attitude shifts by enabling users to see their own experiences mirrored in others, fostering self-efficacy, and normalizing change. This is particularly important in domains involving identity, stigma, or lifestyle transformation, where emotional meaning often precedes behavioral action [60]. Because attitudes involve deeper psychological and social meaning, they usually evolve more slowly than knowledge and may require prolonged engagement in safe, supportive environments before measurable shifts occur.

The differential success of digital features across these outcomes underscores the need for psychological alignment between tool design and intended changes. Knowledge acquisition occurs in simple, repetitive environments. Behavioral changes flourish under real-time reinforcement and structured goal progression. Attitudinal transformation requires social connection, narratives, and emotions. Importantly, intervention duration interacts with these dynamics: knowledge can improve in the short term, but lasting changes in attitude and especially behavior demand extended exposure and reinforcement over time. The most effective interventions addressed all three by combining modalities such as layering peer support on top of informational messaging or embedding reflection within a behavior-tracking platform [61]. These layered approaches enable users to not only learn and act but also internalize and sustain change.

Beyond the distinction across knowledge, attitudes, and behaviors, another layer of explanation lies in the contrast between single-feature and multi-feature interventions. In our review, single-feature tools (e.g., messaging, gamification, or standalone apps) often produced strong but narrow effects, particularly in domains where the psychological demand was relatively simple, such as knowledge acquisition. By contrast, multi-feature interventions such as the integration of wearables with coaching platforms or apps combined with monitoring tools, tended to yield broader and more sustained outcomes, extending their influence beyond knowledge and behavior into attitudinal and psychosocial domains. Similar patterns have been reported in prior reviews, which highlight that digital interventions combining multiple modalities are more effective for complex outcomes such as weight management and chronic disease control than single-feature approaches alone [62]. This pattern is consistent with the COM-B model, in which information-based features primarily enhance capability, social and environmental components create opportunity, and motivational mechanisms such as feedback and accountability help sustain motivation. Taken together, these findings underscore that effectiveness is not an intrinsic property of any single digital feature, but emerges from how features are strategically combined to meet the multifaceted requirements of behavior change.

### 4.4 Practical implication (policy oriented)

This evidence highlights the critical need for policymakers, health program planners, and digital health developers to adopt a targeted and evidence-based approach for selecting digital platforms for public health interventions. Given the diverse impacts associated with different digital features, it is essential that intervention design aligns with the specific health outcomes pursued.

Messaging platforms have emerged as the most versatile and impactful tools, demonstrating high effectiveness in both knowledge acquisition (up to 95 %) and behavioral change (up to 95 %). These platforms are particularly well suited for broad public health campaigns and educational outreach, especially when rapid dissemination of information and reinforcement of key health messages are required [63]. Policymakers should consider investing in a scalable messaging-based infrastructure (SMS services, WhatsApp bots, and Telegram channels) that can support two-way communication, personalization, and real-time engagement [64]. However, such investments must also address the persistent digital divide by ensuring access to affordable devices, stable internet connections, and offline-compatible features for populations in remote or resource-limited settings. Practical lessons can be drawn from successful WhatsApp-based diabetes education programs in low- and middle-income countries, where simple, low-bandwidth solutions proved effective in reaching underserved communities. Although mobile applications and wearable technologies generally yield moderate behavioral improvements, they are best positioned for interventions requiring ongoing behavioral monitoring, self-management, or feedback loops, such as the management of chronic diseases, physical activity, and dietary behaviors. These tools are ideal for long-term interventions where sustained user engagement and behavior tracking are necessary [65]. Public-private partnerships with app developers or health tech firms may facilitate broader access, particularly if interoperability with existing health systems is ensured.

Gamification features showed relatively moderate gains in knowledge (up to 31 %) and strong potential to foster behavior change (up to 93 %), especially among younger populations or individuals who benefited from interactive motivational formats. Thus, policies promoting digital literacy and health gamification could be particularly effective in schools, workplaces, and adolescent health programs [66]. For schools specifically, integration would require alignment with existing curricula, teacher training, and collaboration with ministries of education to ensure that gamified health content is both pedagogically sound and feasible for classroom use. Equally important, the cultural adaptability of digital tools must be considered. Social acceptance and cultural norms play a decisive role in shaping the “opportunity” component of the COM-B framework. For instance, while social media–based interventions may thrive in urban and digitally connected populations, they could encounter resistance in more conservative or rural communities. This highlights the necessity of tailoring digital platforms to the socio-cultural realities of their target groups. Taken together, this evidence suggests that a one-size-fits-all approach to digital health intervention is suboptimal. Instead, national and regional health strategies should encourage context-specific digital designs guided by both the target audience's needs and the desired outcomes (knowledge, attitude, or behavior). From a policy perspective, embedding cultural adaptability into digital health planning would not only enhance effectiveness but also safeguard equity, ensuring that interventions resonate with local values and avoid unintended exclusion. Moreover, digital health policies should emphasize integration, accessibility, and user-centered design, ensuring that interventions are inclusive, sustainable, and aligned with the broader goals of Universal Health Coverage (UHC) and digital equity [67].

In summary, policy frameworks should not only support the deployment of digital tools but also institutionalize mechanisms for the continuous evaluation and adaptation of digital interventions. This would ensure optimal resource utilization, increase the health impact, and improve public trust in digital health innovations.

### 4.5 Incremental contribution

Previous systematic reviews (e.g., Mapping the Landscape of Digital Health Intervention Strategies: 25-Year Synthesis, JMIR 2025; Digital Health Interventions to Improve Access and Quality of Primary Health Care Services, MDPI) have provided broad overviews of digital health strategies, often focusing on strategy typologies, delivery modalities, or access issues across global settings. While valuable, these studies did not systematically disaggregate outcomes by knowledge, attitude, and behavior for different combinations of digital features across target populations (children, adults, elderly) and NCD types in the most recent literature (2022–2024).

The present review builds on and complements these earlier works by specifically examining how platform–population–outcome matchings influence intervention effectiveness. In doing so, it integrates evidence from newer studies (2022–2024) and provides comparative insights into single vs. multi-feature interventions, enabling a more nuanced understanding of what combinations yield stronger improvements across knowledge, attitude, and behavior domains. A comparison table (Supplementary Table 5) highlights these differences: number of studies, outcome domains assessed, and key performance for both existing reviews and the current review.

### 4.6 Study limitations

This systematic scoping review had several limitations that warrant consideration. Although a structured and comprehensive search strategy was employed, the scope was restricted to the selected electronic databases and full-text articles published in English. This may have resulted in the exclusion of potentially relevant studies indexed in the sources or published in languages other than English. While this decision ensured feasibility and consistency of appraisal within the research team's expertise, it also introduced a potential risk of language bias. Additionally, while a formal critical appraisal was conducted to assess the methodological quality of the included studies, the absence of a meta-analytic synthesis limited the ability to quantify effect sizes and compare outcomes across interventions. There was also considerable heterogeneity in study designs, intervention formats, and outcome measures, with some relying on self-reported indicators and others using objective assessments, which complicates direct comparisons. Moreover, most studies assessed only short-term outcomes, leaving the long-term sustainability of behavioral changes uncertain.

Nevertheless, the inclusion of critical appraisal enhances the interpretative value of this review by providing insights into the overall rigor and trustworthiness of available evidence. Furthermore, the effectiveness range in this review was estimated using delta mean values converted into approximate percentage changes. This approach, while practical for comparison purposes, may not fully capture statistical variability or standardized effect sizes. Given the thematic nature of this synthesis, the findings were interpreted qualitatively rather than statistically, which may limit generalizability across contexts. Finally, publication bias and selective reporting within the included studies may have influenced the reported outcomes, particularly, given the predominance of positive intervention effects. Beyond methodological considerations, the generalizability of digital health interventions may also be constrained by unequal access to technology and varying levels of digital literacy, which risk exacerbating health disparities. In addition, the cultural adaptation of digital interventions remains a critical issue, as tools developed in one sociocultural setting may not be directly transferable to others without modification.

## 5 Conclusion

Messaging platforms and mobile apps emerged as the most frequently employed digital interventions, primarily targeting adult and general populations. Each digital feature demonstrated distinct strengths: messaging platforms and gamification were more effective in enhancing knowledge, while mobile apps and wearables were more commonly associated with behavioral improvements. Overall, the choice of digital features influenced both the magnitude and the type of health outcomes achieved, underscoring the importance of aligning platform design with intended behavioral goals. Future studies should explore how combinations of multiple digital features and user engagement strategies can produce synergistic effects on sustained behavior change across diverse populations.

## Location of the institution

Faculty of Public Health, Diponegoro University, Semarang, Indonesia.

## Funding

This study and the publication were supported by the Faculty of Public Health 10.13039/501100005844Diponegoro University.

## Author contributions' statement

ZS was responsible for developing the research background and identifying key gaps in the literature, thereby ensuring a strong conceptual foundation for the study. SAQ refined the methodological framework, including the selection of studies and data extraction process. AK led the synthesis of findings, organized the results, and drew meaningful comparisons. MSM contributed to the critical interpretation of findings, explored their broader implications, and linked them to existing research and practice. All authors collaborate in shaping the final manuscript, revising and refining the content to meet academic standards.

## CRediT authorship contribution statement

**Zahroh Shaluhiyah:** Writing – original draft, Methodology, Funding acquisition, Formal analysis, Conceptualization. **Shabrina Arifia Qatrannada:** Writing – original draft, Project administration, Methodology, Investigation, Formal analysis, Data curation. **Aditya Kusumawati:** Writing – review & editing, Visualization, Validation, Supervision, Resources. **Mohammad Shahgahan Miah:** Writing – review & editing, Visualization, Validation, Software, Resources.

## Declaration of competing interest

The authors declare the following financial interests/personal relationships which may be considered as potential competing interests:

Zahroh Shaluhiyah reports financial support was provided by Faculty of Public Health, Diponegoro University. Zahroh Shaluhiyah reports a relationship with Faculty of Public Health, Diponegoro University that includes: employment and funding grants. If there are other authors, they declare that they have no known competing financial interests or personal relationships that could have appeared to influence the work reported in this paper.

## Appendix A Supplementary data

Supplementary material Image 1
