# Supplementary material for: The effect of digital health intervention in promoting healthy behavior: A systematic scoping review on strategies to prevent non-communicable diseases
Source: Dialogues Health. 2025 Nov 14;7:100258. doi: 10.1016/j.dialog.2025.100258 (PMC12664971; doi:10.1016/j.dialog.2025.100258)
Supplement: Supplementary file 1 — Supplementary material [file mmc1.docx]

**SUPPLEMENTARY FILE**

**Study Selection**

Table 1. Detail process of study selection

| **Step** | **Tools** | **Output** |
| --- | --- | --- |
| Generating synonim | MesH | - social media, social medium, media - health promotion, health campaign, health education, promotion of health - intervention, strategies, procedures - degenerative disease, noncommunicable disease, noninfectious disease, chronic disease |
| Searching related articles | Pubmed | - (((Social Media) AND (Intervention)) AND (Noncommunicable Disease)) AND (Health Promotion) 🡪 **14** - **(((("social media") AND (intervention)) AND ("noninfectious disease)) NOT (systematic review) NOT (scoping review) 🡪 23** |
|  | Sciencedirect | - “health promotion intervention for noncommunicable disease using “social media” 🡪**267 🡪 57 (OR only)** - intervention for noncommunicable disease using “social media” **🡪 171 (OR only)** |
|  | Scopus | - “health promotion” OR “health education” AND intervention OR strategies AND “degenerative disease” OR “noncommunicable disease” OR “noninfectious disease” AND “social media” OR “media social” 🡪 **22** - “health promotion” AND intervention AND Noncommunicable Disease AND “social Media” 🡪 **7 (2018-2023)** - “health education” AND intervention AND Noncommunicable Disease AND “social Media” 🡪 **5 (2015-2023)** |
|  | Jstore | - ((((((("Social Media") AND (Intervention)) AND ("Noncommunicable Disease")) OR ("Noninfectious Disease")) NOT (Review)) AND (Public Health)) OR ("Degenerative Disease")) **🡪 35** - social media intervention for noncommunicable disease **🡪 71** |
|  | Medline | - social media AND intervention OR strategies AND noncommunicable diseases OR degenerative disease NOT ( systematic review or meta analysis ) NOT review 🡪 **3.274 🡪 filter fokus di degenerative and social media intervention 🡪 70** |
|  | Cinahl | - social media AND intervention OR strategies AND noncommunicable diseases OR degenerative disease NOT ( systematic review or meta analysis ) NOT review **🡪 8.399 🡪 128** |
|  | Proquest | - "health promotion" AND intervention AND "noncommunicable diseases" AND "social media" NOT "systematic review" NOT "scoping Review" NOT review **🡪 88** |
|  | Before Filters | **14+23+267+315+22+7+5+3.274 + 8.487 = 12.414** |
|  | Total Article | **691** |
| **Cleaning** | Remove Duplicates | **483** |
|  | Based on Title | - **Cinahl 🡪 40** - **Jstore 🡪 39** - **Medline 🡪 51** - **Proquest 🡪 10** - **Pubmed 🡪 15** - **Sciencedirect 🡪 14** - **Scopus 🡪 11** - **Total 🡪 180** |
|  | Based on Abstract | - **Cinahl 🡪 9** - **Jstore 🡪 3** - **Medline 🡪 6** - **Proquest 🡪 2** - **Pubmed 🡪 7** - **Sciencedirect 🡪 3** - **Scopus 🡪 4** - **Total 🡪 34** |
|  | Based on availability | **20** |

**Critical appraisal**

RCT studies

1. Was true randomization used for assignment of participants to treatment groups?
2. Was allocation to groups concealed?
3. Were treatment groups similar at the baseline?
4. Were participants blind to treatment assignment?
5. Were those delivering the treatment blind to treatment assignment?
6. Were treatment groups treated identically other than the intervention of interest?
7. Were outcome assessors blind to treatment assignment?
8. Were outcomes measured in the same way for treatment groups?
9. Were outcomes measured in a reliable way?
10. Was follow-up complete and, if not, were differences between groups in terms of their follow-up adequately described and analyzed?
11. Were participants analyzed in the groups to which they were randomized?
12. Was appropriate statistical analysis used?
13. Was the trial design appropriate and any deviations from the standard RCT design (individual randomization, parallel groups) accounted for in the conduct and analysis of the trial?

Table 2. Critical appraisal for RCT studies

| **No** | **Author Name and Year** | **Q1** | **Q2** | **Q3** | **Q4** | **Q5** | **Q6** | **Q7** | **Q8** | **Q9** | **Q10** | **Q11** | **Q12** | **Q13** | **Score** | **Responses of Yes** | **Risk of Bias** |
| --- | --- | --- | --- | --- | --- | --- | --- | --- | --- | --- | --- | --- | --- | --- | --- | --- | --- |
| **1** | Vizeshfar (2021) | **Y** | **Y** | **Y** | **N** | **N** | **N** | **U** | **Y** | **Y** | **Y** | **Y** | **N** | **Y** | 8 | 62% | Moderate |
| **2** | Young (2021) | Y | U | Y | U | N | U | U | Y | Y | Y | N | Y | Y | 7 | 54% | Moderate |
| **3** | Schreiber (2023) | Y | U | Y | Y | N | U | U | Y | Y | N | Y | Y | Y | 8 | 62% | Moderate |
| **4** | Hasseldal (2022) | Y | Y | Y | N | N | U | N | N | Y | N | U | Y | Y | 6 | 46% | High |
| **5** | Mack (2020) | Y | N | Y | U | N | N | N | U | Y | N | U | Y | Y | 5 | 38% | High |
| **6** | Frome (2020) | Y | Y | Y | Y | N | Y | N | Y | Y | U | Y | Y | Y | 10 | 77% | Low |
| **7** | Pope (2019) | Y | Y | Y | U | N | U | N | Y | Y | Y | Y | Y | Y | 9 | 69% | Low |
| **8** | Pantes (2021) | Y | Y | Y | U | U | N | N | U | Y | Y | U | Y | Y | 7 | 54% | Moderate |
| **9** | Peuters (2024) | Y | Y | Y | U | N | Y | N | Y | Y | Y | Y | Y | Y | 10 | 77% | Low |
| **10** | Vasil (2023) | Y | N | Y | U | N | U | N | U | Y | Y | Y | Y | Y | 7 | 54% | Moderate |
| **11** | Brammal (2024) | Y | N | Y | U | N | N | N | Y | Y | Y | Y | Y | Y | 8 | 62% | Moderate |
| **12** | Elling (2020) | Y | N | Y | U | N | Y | N | Y | Y | Y | Y | Y | Y | 9 | 69% | Low |

NON RCT studies

1. Is it clear in the study what is the ‘cause’ and what is the ‘effect’ (i.e. there is no confusion about which variable comes first)?
2. Were the participants included in any comparisons similar?
3. Were the participants included in any comparisons receiving similar treatment/care, other than the exposure or intervention of interest?
4. Was there a control group?
5. Were there multiple measurements of the outcome both pre and post the intervention/exposure?
6. Was follow up complete and if not, were differences between groups in terms of their follow up adequately described and analyzed?
7. Were the outcomes of participants included in any comparisons measured in the same way?
8. Were outcomes measured in a reliable way?
9. Was appropriate statistical analysis used?

Table 3. Critical appraisal for Non-RCT studies

| **No** | **Author Name and Year** | **Q1** | **Q2** | **Q3** | **Q4** | **Q5** | **Q6** | **Q7** | **Q8** | **Q9** | **Score** | **Responses of Yes** | **Risk of Bias** |
| --- | --- | --- | --- | --- | --- | --- | --- | --- | --- | --- | --- | --- | --- |
| **1** | Weiner (2023) | Y | N | N | N | Y | Y | Y | Y | Y | 6 | 67% | Moderate |
| **2** | Rahbar (2024) | Y | Y | Y | Y | Y | U | Y | Y | Y | 8 | 89% | High |
| **3** | Alhamzi (2024) | Y | N | N | N | Y | Y | Y | U | Y | 5 | 56% | Moderate |
| **4** | Ashley (2021) | Y | N | N | N | Y | U | Y | N | Y | 4 | 44% | Low |
| **5** | Jarar (2022) | Y | Y | Y | Y | Y | U | Y | N | Y | 7 | 78% | High |
| **6** | Chang (2022) | Y | Y | Y | Y | Y | N | Y | Y | Y | 8 | 89% | High |
| **7** | Lee (2020) | Y | Y | Y | Y | Y | N | Y | Y | Y | 8 | 89% | High |
| **8** | Chang (2022) | Y | Y | Y | Y | Y | Y | Y | U | Y | 8 | 89% | High |

**Relative change (%)**

$$Relative change \left( \% \right)= \frac{Mean after-Mean before}{Mean before} x 100\%$$

Positive values indicate improvement (increase), whereas negative values indicate decline (decrease) in the outcome measure.

Table 4. Specific relative change (%)

| **No** | **Authors** | **Assessment Method** | **Knowledge** | **Attitudes** | **Behavior** |
| --- | --- | --- | --- | --- | --- |
| **1** | Vizeshfar, 2021 (24) | Messaging Platforms |  |  | 4-7% |
| **2** | Young, 2021 (25) | Messaging Platforms |  |  | 12.8% |
| **3** | Weiner, 2023 (26) | Wearable & Monitoring; Video Conferencing Tools |  | 10% | 40% |
| **4** | Rahbar, 2024 (27) | Messaging Platforms |  | 13%-30% | 45% |
| **5** | Schreiber, 2023 (28) | Wearables & Monitoring |  |  | 9% |
| **6** | Alhazmy, 2024 (29) | Messaging Platforms | 95% |  | 25-95% |
| **7** | Ashley, 2021 (30) | Social Media Platforms | 20-40% | 20-40% | 20-40% |
| **8** | Jarrar, 2022 (31) | Messaging Platforms | 45-90% |  |  |
| **9** | Hesseldal, 2022 (32) | Mobile Apps | 40% |  |  |
| **10** | Chang, 2022 (33) | Gamification | 10% |  |  |
| **11** | Mack, 2020 (34) | Gamification | 30% |  |  |
| **12** | Froome, 2020 (35) | Gamification | 31% |  |  |
| **13** | Pope, 2019 (36) | Wearables & Monitoring; Social media platforms |  | 25-35% | 25-35% |
| **14** | Pontes, 2021 (37) | Gamification |  |  | 40-93% |
| **15** | Peuters, 2024 (38) | Mobile Apps |  |  | 5-30% |
| **16** | Vasil, 2023 (39) | Gamification; Mobile apps |  |  | 9-24% |
| **17** | Brammall, 2024 (40) | Web-based Tools, Video Conferencing |  |  | 35-64% |
| **18** | Lee, 2020 (41) | Mobile Apps; Wearables & Monitoring |  | 17% | 11% |
| **19** | Chang, 2022 (43) | Messaging Platforms |  | 2-10% |  |
| **20** | Elling, 2020 (44) | Web-based Tools | 31% | 31% |  |

**Comparison of existing studies**

Table 5. Comparison of existing systematic reviews and the present review

| **Review (Author, Year)** | **Literature Coverage** | **Primary Focus** | **Population/Setting** | **Outcome Domains** | **NCD-Specific?** | **Comparative Insights** |
| --- | --- | --- | --- | --- | --- | --- |
| ***Mapping the Landscape of Digital Health Intervention Strategies: 25-Year Synthesis* (JMIR, 2025)** | 2000–2022 | Strategy typologies, delivery modalities, access | Global, mixed populations | Broad health outcomes (not disaggregated) | No | Valuable synthesis of strategies but lacks outcome breakdown (knowledge, attitude, behavior) |
| ***Digital Health Interventions to Improve Access and Quality of Primary Health Care Services* (MDPI, 2025)** | 2000–2023 | Access and quality of care improvement | Global, primary health care settings | Service access, quality metrics | Partially | Highlights access barriers and delivery models but does not map digital features to outcome domains |
| **Present Review (2025)** | 2022–2024 | Platform–population–outcome matchings; single vs. multi-feature comparisons | Children, adults, elderly (NCD focus) | Knowledge, Attitude, Behavior (systematically disaggregated) | Yes (NCD-focused) | Provides comparative insights into effectiveness of digital feature combinations; highlights nuanced outcome patterns |
